# Fear of childbirth and elective caesarean section: a population-based study

**DOI:** 10.1186/s12884-015-0655-4

**Published:** 2015-09-17

**Authors:** Hege Therese Størksen, Susan Garthus-Niegel, Samantha S. Adams, Siri Vangen, Malin Eberhard-Gran

**Affiliations:** Health Services Research Centre, Akershus University Hospital, Post Box 1000, 1478 Lørenskog, Norway; Institute of Clinical Medicine, Campus Ahus, University of Oslo, Lørenskog, Norway; Institute and Policlinic of Occupational and Social Medicine, Faculty of Medicine, TU Dresden, Dresden, Germany; Norwegian Resource Centre for Women’s Health, Women and Children’s Division, Oslo University Hospital, Rikshospitalet, Oslo, Norway; Department of Obstetrics and Gynaecology, Akershus University Hospital, Lørenskog, Norway; Division of Mental Health, Norwegian Institute of Public Health, Oslo, Norway; Division of Epidemiology, Norwegian Institute of Public Health, Oslo, Norway

## Abstract

**Background:**

This population-based cohort study aimed to investigate the demographic and psychosocial characteristics associated with fear of childbirth and the relative importance of such fear as a predictor of elective caesarean section.

**Methods:**

A sample of 1789 women from the Akershus Birth Cohort in Norway provided data collected by three self-administered questionnaires at 17 and 32 weeks of pregnancy and 8 weeks postpartum. Information about the participants’ childbirths was obtained from the hospital records.

**Results:**

Eight percent of the women reported fear of delivery, defined as a score of ≥85 on the Wijma Delivery Expectancy Questionnaire. Using multivariable logistic regression models, a previous negative overall birth experience exerted the strongest impact on fear of childbirth, followed by impaired mental health and poor social support. Fear of childbirth was strongly associated with a preference for elective caesarean section (aOR 4.6, 95 % CI 2.9–7.3) whereas the association of fear with performance of caesarean delivery was weaker (aOR 2.4, 95 % CI 1.2–4.9). The vast majority (87 %) of women with fear of childbirth did not, however, receive a caesarean section. By contrast, a previous negative overall birth experience was highly predictive of elective caesarean section (aOR 8.1, 95 % CI 3.9–16.7) and few women without such experiences did request caesarean section.

**Conclusions:**

Results suggest that women with fear of childbirth may have identifiable vulnerability characteristics, such as poor mental health and poor social support. Results also emphasize the need to focus on the subjective experience of the birth to prevent fear of childbirth and elective caesarean sections on maternal request. Regarding the relationship with social support, causality has to be interpreted cautiously, as social support was measured at 8 weeks postpartum only.

## Background

Childbirth is one of the most important events in a woman’s life. Parturition is the transition to motherhood and delivery has substantial physical and emotional impacts. Approximately 6 to 10 % of all pregnant women experience severe fear of childbirth [1–3]. This fear may be the dominant emotion during pregnancy and may complicate and prolong labour [4]. Moreover, severe fear of childbirth may lead to increased risk of postnatal depression [5], and posttraumatic stress disorder [6]. Numerous factors have been associated with that fear, including low self-esteem, pre-existing psychological problems, lack of social support, a history of abuse, or a previous negative birth experience [3,7–11]. It is conceivable that demographic and psychosocial factors may increase stress related to impending childbirth and are connected with the ways women anticipate and experience various life events. Consequently, those characteristics could be predictive of fear of childbirth. However, few studies have focused on the relative importance of both demographic and psychosocial factors [7, 12], and several studies of fear of childbirth and its association with these factors have been limited by a small sample size [10] or use of non-validated questions [7] or other unspecific measurements [12].

Another important issue is the increasing number of women who deliver by caesarean section (CS) in almost all countries in the western world over the last 30 years [13, 14]. Although Norway has a relatively low caesarean section rate compared to other European countries, rates have increased from 2.5 % in 1972 [13] to 17 % in 2011 (Norwegian Institute of Public Health website, 2014). Currently, the proportion of all caesarean deliveries that are elective in Norway varies between 30 and 47 % (Norwegian Institute of Public Health website, 2014). The majority of caesarean sections are performed for medical reasons; however, an increasing number are performed as a result of maternal request without a medical indication. This development is concerning because a caesarean section without medical indication may not confer health gain, can result in dangerous side effects, and is more costly than vaginal deliveries. When categorized by cause, 14–22 % of all elective caesareans in Norway are performed upon maternal request [13].

Several studies has shown that fear of childbirth often is an underlying factor for a mother’s request for caesarean section [9, 15, 16]. Hence, childbirth-related anxiety has been suggested to be a main reason for the increase in elective caesarean sections [17, 18]. Fear of childbirth might affect women in such a way that they begin to doubt themselves and feel uncertain of their ability to bear and give birth to a child [19]. Although an association between fear of childbirth and a request for caesarean section has previously been shown, few studies have assessed the association between fear of childbirth and performance of elective caesarean section [12]. In particular, no previous study has assessed the independent effect of fear on the elective caesarean section rate taking medical risk factors and previous overall birth experiences into account.

The aim of this study was to investigate (a) the demographic and psychosocial characteristics associated with fear of childbirth and (b) the relative importance of such fear on both caesarean delivery preference and delivery by elective caesarean section among 1789 pregnant Norwegian women.

## Methods

### Patients

The study sample was drawn from the Akershus Birth Cohort Study, which targeted all women scheduled to give birth at Akershus University Hospital, Norway [11]. The hospital is located near Oslo, the capital of Norway, and serves a total population of approximately 400,000 individuals from both urban and rural surroundings. On average, 3500 women gave birth each year at the hospital during the study period.

**Fig. 1:**
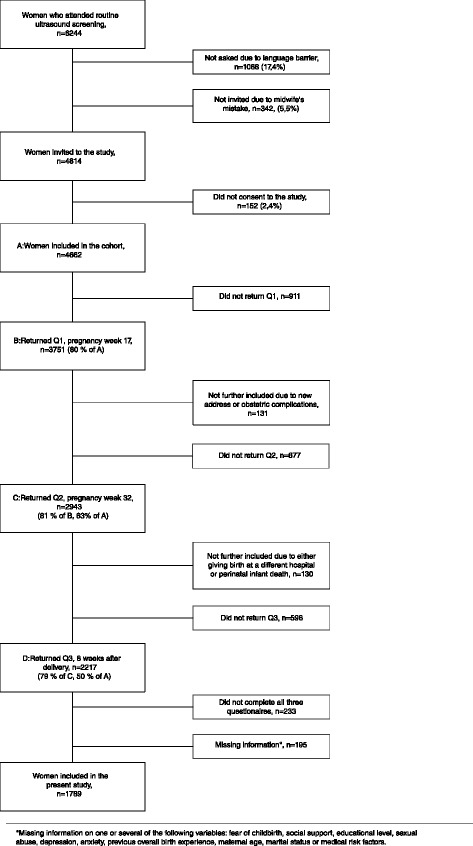
Study flow chart

Women were recruited at a routine fetal ultrasound examination in pregnancy week 17 from November 2008 to April 2010. As part of the public antenatal care program, this examination is offered free of charge to all women in the hospital’s catchment area. Pregnant women who were able to complete a questionnaire in Norwegian were eligible for the Akershus Birth Cohort Study. A total of 4662 women were included in the cohort (Fig. 1). Of these, 80 % (*n* = 3751) returned the first questionnaire. Participants also completed questionnaires at pregnancy week 32 and 8 weeks postpartum, with response rates of 81 % (2943 out of 3620) and 79 % (2217 out of 2813), respectively. A total of 1984 women answered all three questionnaires and comprised our baseline sample. Additional information on the pregnancies and births was obtained by linkage to the electronic birth records for the obstetric ward. The birth records were completed by the doctor or midwife in charge of the delivery. We excluded women with records that were missing information on fear of childbirth (*n* = 31), social support (*n* = 31), educational level (*n* = 81), sexual abuse (*n* = 4), depression (*n* = 3), anxiety (*n* = 5), previous overall birth experience (*n* = 8), maternal age (*n* = 14), marital status (*n* = 27), and medical risk factors (*n* = 14). This resulted in a final study sample of 1789 women (some women had missing information on several variables).

All women asked to participate were given written information explaining the purpose of the study and were informed that participation was voluntary. Informed consent was obtained from all participants. The study was approved by the Regional Committee for Ethics in Medical Research in Norway, approval number S-08013a.

### Measures

#### Fear of childbirth

**Fig. 2:**
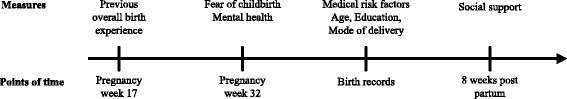
Data collection, points of time

Fear of childbirth was assessed with the Wijma Delivery Expectancy/Experience Questionnaire version A (W-DEQ) at pregnancy week 32 (Fig. 2). The W-DEQ is a 33-item self-assessment rating scale, and each response is rated on a six-point Likert scale, ranging from 0 to 5 [20]. Therefore, summed scores may range from 0 to 165, with higher scores reflecting a greater degree of fear of childbirth. In the data analyses, fear of childbirth was defined as a W-DEQ total score ≥85. This cut-off has been commonly used to distinguish between women with and those without fear of childbirth [21]. Details regarding the Norwegian version of the W-DEQ are described elsewhere [11].

#### Social support

Social support during pregnancy was measured 8 weeks after delivery with the three-item Oslo Social Support Scale. The scale has been used in several studies that confirm its feasibility and predictive validity with respect to psychological distress [22, 23]. The total score is calculated by summing the individual item scores. Summed scores may range from 3 to 14, with the following categories: 3–8 = poor support, 9–11 = moderate support and 12–14 = strong support [24, 25].

#### Sexual abuse

The history of sexual abuse was assessed with an adapted version of the Abuse Assessment Screen [26] at 8 weeks postpartum. The questions were: “Have you ever, as an adult, been coerced into sexual activities?” (no/yes) and “Have you ever, as an adult, been forced into sexual activities?” (no/yes). The answers were coded as no/coerced/forced. While there is currently no gold standard for measuring sexual abuse, the Abuse Assessment Screen has previously been shown to be valid, and is comparable to several more extensive measures [26].

#### Depression

Symptoms of depression during the past week were measured in pregnancy week 32 with the Edinburgh Postnatal Depression Scale (EPDS) [27, 28]. The EPDS is a 10-item self-rating scale designed to identify symptoms of depression after delivery. The scoring of each item ranges from 0 (absence of symptoms) to 3 (maximum severity of symptoms) [27]; thus, the sum EPDS score ranges from 0 to 30. In the data analyses, depression was defined as an EPDS score ≥12 [27, 28]. The scale has been validated for detection of major and minor depression in pregnant women [29], postpartum women [27], and non-postpartum women [28]. The Norwegian version of the EPDS has been validated against the DSM-IV criteria (Diagnostic and Statistical Manual of Mental Disorders, 4th edition) for major depression [30].

#### Anxiety

Symptoms of anxiety during the past week were measured in pregnancy week 32 with the first 10 items in the Hopkins Symptom Check List (SCL-25) [31, 32]. The SCL-25 is a widely used self-rating scale, and the first 10 items comprise the anxiety score (SCL-anxiety). Each item ranges from ‘not at all’ (score 1) to ‘extremely’ (score 4), and the sum score for anxiety may range from 10 to 40. Presence of anxiety was defined as SCL-anxiety score ≥18 [33–35]. The SCL-25 was designed to measure symptoms of depression and anxiety and has been extensively used in population-based questionnaires in Norway (website of Statistics Norway: http://www.ssb.no). The Norwegian version of the SCL-25 has been validated against the ICD-10 criteria (International Classification of Diseases, 10th edition) for anxiety and depression [36].

#### Medical risk factors

Information on medical risk factors was retrieved from the maternity ward birth records. Each risk factor was treated as a dichotomous variable, depending on whether or not it was present during pregnancy. The risk factors recorded included: (1) heart disease, (2) chronic hypertension, (3) chronic kidney disease, (4) asthma, (5) epilepsy, (6) rheumatoid arthritis, (7) diabetes, (8) gestational hypertension, (9) preeclampsia before week 34, (10) twins, (11) non-cephalic presentation, (12) large foetus (>4500 g), and (13) previous delivery by caesarean section. A previous delivery by caesarean section increases the risk of recurrent caesarean section [13, 17], and was therefore included as a medical risk factor. The medical risk factors were coded as none, one risk factor, or two or more.

#### Previous overall birth experience

Information on previous overall birth experience was assessed from the first questionnaire at pregnancy week 17 and measured using a numeric rating scale (NRS). The NRS was based on the question: “What was your overall experience of the birth?” The answers were scored from 0 (very good) to 10 (extremely bad). We defined a previous negative overall birth experience as an NRS score ≥9, roughly representing the upper 10th percentile. Primiparas were grouped as ‘women with no previous delivery experience’.

#### Other variables

Information on maternal education, age at delivery, marital status, and mode of delivery was obtained from the maternity ward birth records. Years of education was coded as: ≤12 or >12, maternal age at delivery was coded as: ≤ 25; 26–35; ≥ 36, and marital status was coded as: married or cohabiting, or single. Mode of delivery was categorized as an elective caesarean delivery or as another delivery mode; including vaginal, instrumental vaginal (vacuum or forceps-assisted delivery) or acute caesarean delivery. The term elective caesarean delivery included caesarean deliveries planned 8 h or more before delivery and performed as planned. Preference for an elective caesarean section was based on the following question at 32 weeks of gestation: “If I could choose, I would prefer to deliver by caesarean section.” The answers were coded as: yes (highly agree/agree) or no (disagree/highly disagree).

### Statistical methods

The prevalence (%) of fear of childbirth, preference for elective caesarean section, and delivery by elective caesarean section were calculated. Differences in the distribution of categorical study factors according to fear of childbirth, preference for elective caesarean section, and delivery by elective caesarean section were tested with chi-squared tests and are presented as proportions (%). The associations between demographic or psychosocial study factors and the fear of childbirth were estimated in terms of crude and adjusted odds ratios (ORs) with 95 % confidence intervals (CIs), based on logistic regression analyses. In addition, the association between fear of childbirth and the preference for elective caesarean section or actual delivery by elective caesarean section was estimated in terms of the adjusted ORs with 95 % CIs, based on logistic regression analyses. A 5 % significance level was chosen. The statistical package SPSS version 15.0 was used for the analyses.

## Results

The mean age of the women was 31 years (range 18–45 years; SD 4.6 years). Forty-nine percent of the women were primiparas. Ninety-nine percent of the women were married or cohabiting with the child’s father. The mean W-DEQ score was 57 (range 2–145; SD 19.5). Eight per cent of the women (134/1789) reported fear of childbirth, defined as W-DEQ score ≥85. In the study sample, 75 % of the women had a normal vaginal delivery, 11 % had an instrumental vaginal delivery, 9 % delivered by emergency caesarean section and 5 % delivered by elective caesarean section.

### Factors associated with fear of childbirth

Fear of childbirth was observed among 16 % (27/169) of women with poor social support, 33 % (24/73) of women with combined anxiety and depression, and 28 % (20/72) of women with a previous negative overall birth experience. Based on binary logistic regression, we found that a previous negative overall birth experience (crude OR 8.4, 95 % CI 4.6–15.5) and combined anxiety and depression (crude OR 8.4, 95 % CI 4.9–14.4) were the most important correlates of the fear of childbirth; these were followed by poor social support (crude OR 6.1, 95 % CI 3.4–11.0) and no previous delivery experience (crude OR 2.1, 95 % CI 1.4–3.2) (Table 1). After mutual adjustment for the other study factors in a logistic regression model, a previous negative overall birth experience remained the strongest factor for fear of childbirth (aOR 7.6, 95 % CI 3.8–15.2), followed by combined anxiety and depression (aOR 6.1, 95 % CI 3.3–11.2) and poor social support (aOR 3.8, 95 % CI 1.9–7.6). Giving birth for the first time and a high educational level were also associated with fear of childbirth (Table 1).

**Table 1:** Crude and adjusted odds ratio (OR) with 95 % confidence intervals (CI) for fear of childbirth (W-DEQ score ≥85) among 1789 pregnant Norwegian women

	Fear of childbirth (W-DEQ)					
	Low score (<85)	High score (≥85)	Total	*P*	Crude OR	Adjusted OR
	*n* (%)	*n* (%)			(95 % CI)	(95 % CI)
Social support						
Strong	703 (97.0)	22 (3.0)	725 (100)	<0.001	1.0	1.0
Moderate	810 (90.5)	85 (9.5)	895 (100)		3.4 (2.1–5.4)***	3.1 (1.9–5.0)***
Poor	142 (84.0)	27 (16.0)	169 (100)		6.1 (3.4–11.0)***	3.8 (1.9–7.6)***
Educational level (years)						
≤ 12	1137 (92.1)	98 (7.9)	1235 (100)	0.286	1.0	1.0
> 12	518 (93.5)	36 (6.5)	554 (100)		1.2 (0.8–1.8)	2.1 (1.3–3.3)***
Sexual abuse						
No	1390 (93.3)	100 (6.7)	1490 (100)	0.018	1.0	1.0
Coerced	188 (89.1)	23 (10.9)	211 (100)		1.7 (1.1–2.7)*	1.2 (0.7–2.0)
Forced	77 (87.5)	11 (12.5)	88 (100)		2.0 (1.0–3.9)*	0.9 (0.4–2.0)
Mental health						
No mental impairment	1498 (94.5)	87 (5.5)	1585 (100)	<0.001	1.0	1.0
Anxiety or depression	108 (82.4)	23 (17.6)	131 (100)		3.7 (2.2–6.0) ***	3.0 (1.7–5.1)***
Both anxiety and depression	49 (67.1)	24 (32.9)	73 (100)		8.4 (4.9–14.4)***	6.1 (3.3–11.2)***
Medical risk factors						
None	1319 (92.8)	103 (7.2)	1422 (100)	0.715	1.0	1.0
One	287 (91.7)	26 (8.3)	313 (100)		1.2 (0.7–1.8)	1.1 (0.7–1.8)
Two or more	49 (90.7)	5 (9.3)	54 (100)		1.3 (0.5–3.4)	1.2 (0.4–3.6)
Previous overall birth experience						
Good	808 (95.6)	37 (4.4)	845 (100)	<0.001	1.0	1.0
Bad	52 (72.2)	20 (27.8)	72 (100)		8.4 (4.6–15.5)***	7.6 (3.8–15.2)***
No previous delivery experience	795 (91.2)	77 (8.8)	872 (100)		2.1 (1.4–3.2)***	2.3 (1.5–3.5)***

The following variables were also included in the logistic regression analyses, but not significantly associated with fear of childbirth and therefore not included in the table; age of the woman and marital status. *Statistically significant at 0.05 level, *** at 0.001 level. *OR* odds ratio, *CI* confidence interval

### Factors associated with elective caesarean section

**Fig. 3:**
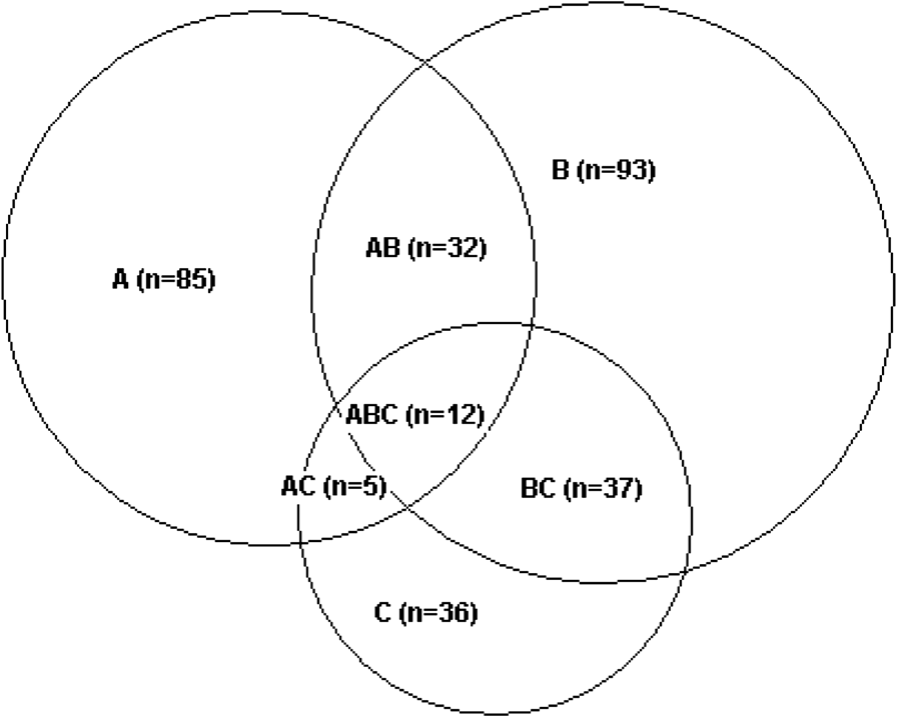
The number of women with fear of childbirth (**a**), preference for caesarean section (**b**) and delivery by elective caesarean section (**c**) among 1789 pregnant women in the Akershus Birth Cohort Study

In the study sample, 9.7 % (174/1789) of the women had a preference for caesarean delivery, and 5 % (90/1789) delivered by elective caesarean section. Among women with a fear of childbirth, 32.8 % (44/134) had a preference for elective caesarean section, and 12.7 % (17/134) delivered by elective caesarean section. Fear of childbirth was strongly associated with preference for caesarean delivery (aOR 4.6, 95 % CI 2.9–7.3) whereas the association with delivery by elective caesarean section was weaker (aOR 2.4, 95 % CI 1.2–4.9) (Table 2). Medical risk factors exerted the strongest impact on delivery by elective caesarean section, with an aOR of 14.3 (95 % CI 8.3–24.8) for one medical risk factor and an aOR of 21.6 (95 % CI 9.6–48.7) for two or more medical risk factors. In addition, a previous negative birth experience was strongly associated with delivery by elective caesarean section (aOR 8.1, 95 % CI 3.9–16.7). Interestingly, women with a high educational level were less likely to have a preference for elective caesarean section than women with a low to moderate educational level. Finally, the majority (87 %, 117/134) of women with fear of childbirth did not undergo an elective caesarean section (Fig. 3).

**Table 2:** Prognostic factors for preference for elective caesarean section (CS) and delivery by elective CS among 1789 pregnant Norwegian women

		Preference for elective CS		Delivery by elective CS	
Variables	No. persons	Cases (%)	Adjusted OR	Cases (%)	Adjusted OR
			(95 % CI)		(95 % CI)
Fear of childbirth week 32 (W-DEQ)					
Low score (<85)	1655	130 (7.9)	1.0	73 (4.4)	1.0
High score (≥85)	134	44 (32.8)	4.6 (2.9–7.3)***	17 (12.7)	2.4 (1.2–4.9)*
Social support					
Strong	725	58 (8.0)	1.0	30 (4.1)	1.0
Moderate	895	94 (10.5)	1.1 (0.8–1.6)	48 (5.4)	1.4 (0.8–2.4)
Poor	169	22 (13.0)	1.0 (0.6–1.9)	12 (7.1)	2.0 (0.9–4.6)
Educational level (years)					
≤ 12	554	72 (13.0)	1.0	35 (6.3)	1.0
> 12	1235	102 (8.3)	0.6 (0.4–0.9)**	55 (4.5)	0.9 (0.5–1.5)
Maternal age (years)					
≤ 25	177	20 (11.3)	1.0	7 (4.0)	1.0
26–35	1269	116 (9.1)	1.0 (0.6–1.7)	56 (4.4)	0.9 (0.4–2.2)
≥ 36	343	38 (11.1)	1.3 (0.7–2.5)	27 (7.9)	1.6 (0.6–4.4)
Sexual abuse					
No	1490	132 (8.9)	1.0	71 (4.8)	1.0
Coerced	211	28 (13.3)	1.4 (0.9–2.3)	11 (5.2)	0.9 (0.4–1.9)
Forced	88	14 (15.9)	1.0 (0.5–2.0)	8 (9.1)	0.7 (0.3–1.8)
Mental health					
No mental impairment	1585	136 (8.6)	1.0	75 (4.7)	1.0
Anxiety or depression	131	24 (18.3)	1.7 (1.0–2.9)*	8 (6.1)	0.9 (0.4–2.2)
Both anxiety and depression	73	14 (19.2)	1.1 (0.5–2.2)	7 (9.6)	0.9 (0.3–2.5)
Medical risk factors					
None	1422	107 (7.5)	1.0	21 (1.5)	1.0
One	313	53 (16.9)	2.4 (1.6–3.5)***	56 (17.9)	14.3 (8.3–24.8)***
Two or more	54	14 (25.9)	4.3 (2.2–8.7)***	13 (24.1)	21.6 (9.6–48.7)***
Previous overall birth experience					
Good	845	63 (7.5)	1.0	38 (4.5)	1.0
Bad	72	26 (36.1)	4.1 (2.2–7.4)***	23 (31.9)	8.1 (3.9–16.7)***
No previous delivery experience	872	85 (9.7)	1.4 (0.9–2.0)	29 (3.3)	1.0 (0.6–1.7)

The associations are estimated as adjusted odds ratios with 95 % confidence intervals. All study factors are mutually adjusted in the logistic regression model. Marital status was also included in the logistic regression analyses, but not significantly associated with preference for elective caesarean section or delivery by elective caesarean section and therefore not included in the table. *Statistically significant at 0.05 level, ** at 0.01 level, *** at 0.001 level. *OR* odds ratio, *CI* confidence interval

## Discussion

In this study of 1789 pregnant women, we aimed to investigate the demographic and psychosocial characteristics associated with fear of childbirth, as well as such fear’s relative importance on both caesarean delivery preference and performance of elective caesarean section.

The key findings are as follows: (1) Women with fear of childbirth were more likely to have had a previous negative overall birth experience, impaired mental health, or poor social support; indicating certain vulnerabilities in these women. (2) Though fear of childbirth was associated with both the preference for and delivery by elective caesarean section, the vast majority of women (87 %) with fear of childbirth did not deliver by elective caesarean section. (3) The main predictors of delivery by elective caesarean section were medical risk factors and a previous negative overall birth experience.

In a previous study, we showed that the majority of women (77.5 %) who experienced a severe obstetric complication did not consider the birth to have been a negative experience [37]. As such, a woman’s experience of giving birth is not necessarily associated with the course of obstetric events. By contrast, another study has shown that a negative birth experience to a large extent can be related to quality of care and/or lack of support women receive during childbirth [19]. In the present study, a previous negative birth experience was the factor most strongly associated with an increased risk of developing fear of childbirth, as well as an increased risk of undergoing an elective caesarean section. Since these associations remained strong after controlling for other relevant risk factors, subjective birth experience is crucial, as is providing women with a positive childbirth experience. To our knowledge, only one other study has explored the independent effect of previous subjective birth experiences on the fear of childbirth [37]. Earlier research has instead focused on obstetric factors [3, 38, 39], whether a negative birth experience derived from fear of childbirth [21, 40, 41], and possible factors of the variation in women’s overall assessment of the birth experience [42, 43].

Maternal psychopathology is a well-known risk factor for fear of childbirth [7, 10, 11, 44–46]. The results of this study also show an association between poor social support and fear of childbirth, which is consistent with previous findings [7, 10].

Along with these psychosocial factors, this study found that giving birth for the first time and a high educational level were factors associated with fear of childbirth. In contrast, the results from a recent study indicate that multiparous women fear childbirth more than first-time mothers [12]. However, their estimates were based on the ICD-10 code O99.8 (‘other specific diseases and conditions complicating pregnancy, childbirth, or puerperium’), which is a rather unspecific measure for fear of childbirth. Other studies have shown that first-time mothers tend to be slightly more anxious than women who have previously given birth [2, 47]. Unlike our results, a high educational level has previously been associated with less fear of childbirth [10, 48]. Compared to our study, those studies included relatively few women. It is conceivable that women with higher levels of education may seek out birth-related information more actively than women with less education and that information about potential risks could engender greater fear of the upcoming delivery.

Several studies have shown that fear of childbirth often underlies a mother’s request for caesarean section [9, 16, 17, 49]. Results of the present study support this finding, since fear of childbirth was the factor most strongly associated with a preference for elective caesarean section. However, fear of childbirth had a somewhat weaker impact on performance of elective caesarean delivery. These results may partly stem from the hospital’s having an anti-fear program for women with fear of childbirth that aim to alleviate those fears and prevent caesarean sections. Such treatment programs focusing on self-perceived fear of childbirth have been implemented at several Norwegian hospitals.

To our knowledge, ours is the first study to investigate the independent effect of fear of childbirth on delivery by elective caesarean section. Although fear of childbirth had a strong impact on a mother’s request for elective caesarean section, other factors determined whether a caesarean section was performed. Medical risk factors were the primary predictors of delivery by elective caesarean section followed by a previous negative overall birth experience. This study’s findings thus show that most women who delivered by elective caesarean section had somatic and/or medical reasons for undergoing the procedure. Our findings also confirm the results from a previous study indicating that few first-time mothers or parous women without previous negative birth experiences request caesarean section [50]. As such, it is possible that not fear, but greater maternal age, more twin pregnancies, and other factors that increase the risk of adverse birth outcomes account for the increased rate of caesarean delivery [51]. Such increase could also be ascribed to changes in clinical management [52], as well as a lower threshold among obstetricians for performing an operative delivery [53, 54].

### Study limitations

This study has both strengths and limitations that should be recognized. Its high response rate and inclusion of women who attended routine antenatal care prevented any bias due to selecting from a group participating in specialized treatment programs. This is important, because most women who fear childbirth are not included in such programs. Plus, given this study’s access to medical records and maternity ward birth records, information regarding mode of delivery and medical risk factors supported the prospective design, which in turn support a relationship between fear of childbirth and elective caesarean section in a causal direction. Furthermore, in contrast to other studies [3, 41, 42, 55, 56], fear of childbirth was measured with a validated psychometric instrument designed to measure fear of childbirth, the W-DEQ [20, 45, 57]. The original validation study showed that the W-DEQ has good internal consistency, with a Cronbach’s alpha coefficient of 0.93 [20]. In the current study, the Cronbach’s alpha coefficient was 0.92. Moreover, the EPDS and SCL-anxiety are validated screening instruments used to identify women with probable depression and anxiety [30, 36]. To our knowledge, however, no established, validated instrument is currently available for measuring previous overall birth experience. Consequently, we used a numeric one-item scale shown to be reliable and valid for measurements of pain, mood, and other subjective feelings [58]. Previous overall birth experiences were measured in pregnancy week 17, and social support was measured by retrospective questioning eight weeks postpartum, both of which may have contributed to a recall bias in some women. However, previous research indicates that social support tends to remain stable over time and across situations, even in periods of developmental change [59]. Nevertheless, some research has shown that a traumatic birth may compromise relationships and could consequently affect the stability of the measure [60, 61]. Therefore, regarding social support, the potential reverse causality has to be kept in mind.

The prevalence of elective caesarean section in the Akershus Birth Cohort Study (5.0 %) was slightly lower compared to that shown by national data from the Medical Birth Registry of Norway from 2009 (6.5 %) (Norwegian Institute of Public Health website, 2014) (Table 3). Nevertheless, the generalizability of the results of this study may be limited by the fact that only Norwegian-speaking women were included, which resulted in a relatively homogeneous, almost entirely Caucasian sample. Different results might be obtained for other ethnic groups. Furthermore, there is reason to believe that the women in the study were somewhat more resourceful than the general birthing population in Norway. Compared to national data from the Medical Birth Registry of Norway, participants in the Akershus Birth Cohort Study were older, more often first time mothers, and less likely to smoke than non-participants (Table 3). However, it is important to bear in mind that selection bias does not necessarily influence results much when associations between variables are investigated [62]. Lastly, though numerous confounders were controlled for, other confounders, which we did not measure, could possibly play a role.

**Table 3:** Comparison of women included in the Akershus Birth Cohort and all women who gave birth in Norway in 2010

	The Akershus Birth Cohort Study	All woman who gave birth in Norway in 2010
Mean maternal age	31.3 years	30.2 years
First time mothers	48.9 %	42.6 %
Smoking during pregnancy	6.6 %	7.2 %
Single women	1.1 %	9.1 %
Elective caesarean section	5.0 %	6.5 %

## Conclusions

Our results suggest that women with fear of childbirth are more likely to have had a previous negative overall birth experience, impaired mental health, and poor social support, which indicates certain vulnerabilities in these women. Fear of childbirth exerted a strong impact on the preference for elective caesarean section, as well as a somewhat weaker impact on performance of elective caesarean section. The vast majority of women with fear of childbirth did not, however, receive a caesarean section.

A previous negative birth experience was strongly predictive of elective caesarean section, and few women without such experiences did request caesarean section. Hence, our results emphasize the need to focus on the subjective experience with the goal of providing women with a positive birth experience. Medical complications can not always be avoided; if they occur, it is important to make women feel safe and cared for. At the same time, a woman may perceive an uncomplicated birth as being traumatic, particularly if she feels that the staff is under stress. Recognizing the significance of the subjective birth experience opens up important opportunities for prevention of fear of childbirth and caesarean section, since subjectively negative birth experiences are, to a large extent, preventable [63]. Further studies are warranted to replicate and extend our findings. Particular attention should also be given to the importance of different health care indicators as predictors of overall birth experience.
